# Associations Between Potential *Listeria monocytogenes* Exposure During Pregnancy and Infant Outcomes at Birth and Infant Delivery Resource Use: A Cross‐Sectional Analysis in Australian Women

**DOI:** 10.1155/jp/7824916

**Published:** 2026-07-31

**Authors:** Kee June Ooi, Sasha Fenton, Rachael Taylor, Madeleine Hinwood, Poonam Kaur Pannu, Melinda Hutchesson, Clare E. Collins

**Affiliations:** ^1^ School of Health Sciences, College of Health, Medicine and Wellbeing, University of Newcastle, Callaghan, New South Wales, Australia, ncl.ac.uk; ^2^ Nutrition and Metabolic Health Research Program, Hunter Medical Research Institute, New Lambton Heights, New South Wales, Australia, hmri.com.au; ^3^ Data Science, Hunter Medical Research Institute, New Lambton Heights, New South Wales, Australia, hmri.com.au; ^4^ School of Medicine and Public Health, College of Health, Medicine and Wellbeing, University of Newcastle, Callaghan, New South Wales, Australia, ncl.ac.uk; ^5^ The Kids Research Institute Australia, Nedlands, Western Australia, Australia; ^6^ Curtin University, Bentley, Western Australia, Australia, curtin.edu.au

**Keywords:** birth outcomes, hospital resource use, infant outcomes, *Listeria monocytogenes*, listeriosis, ORIGINS

## Abstract

Pregnant women are at higher risk of contracting listeriosis, which can lead to serious perinatal complications. This study evaluated associations between *Listeria monocytogenes* exposure, infant perinatal outcomes, and hospital resource use in a cohort of 1604 Australian mother–infant dyads. Maternal *L. monocytogenes* exposure was estimated from self‐reported intake of foods from a validated food frequency questionnaire that potentially harbor *L. monocytogenes*. Infant outcomes obtained from hospital medical records included birth mode, preterm birth, birthweight, and admission to special care nursery (SCN)/neonatal intensive care unit (NICU). Infant hospital resource use was measured by infant length of stay (LOS), LOS in SCN/NICU, and days excluding SCN/NICU. Multinomial, negative binomial, and hurdle models, were performed to examine *Listeria* Food Exposure Score (LFES) associations with infant outcomes, total LOS, and LOS excluding and within SCN/NICU. All models were adjusted for covariates including smoking, parity, maternal age, BMI, and SEIFA IRSAD decile. Mean (SD) maternal age was 32.0 (5.0) years, and median (IQR) gestation was 39.0 (38.1, 40.0) weeks. Although adjusted results showed a statistically significant association between LFES and reduced infant LOS excluding SCN/NICU (*β* = 0.99; 95% CI: 0.979, 0.998, *p* < 0.03), the effect size was minimal, with minor clinical significance. There were no significant associations with infant birth mode, preterm birth, low birthweight, size for gestational age, macrosomia, admission to NICU/SCN, total LOS in hospital, and SCN/NICU (all *p* > 0.05). Future research should explore these associations among ethnically diverse women at earlier stage of pregnancy and include the assessment of food safety practices in the analyses.

## 1. Introduction

Listeriosis is a foodborne illness that can be acquired by consuming foods contaminated with the bacterium, *Listeria monocytogenes* [1]. This bacterium can survive at refrigeration temperature (0°C–5°C) [2], posing a risk of contamination to various food products such as delicatessen meat, soft cheeses, pre‐prepared fruits, and salads [3]. Listeriosis can be categorized into two types: non‐invasive and invasive [4]. Non‐invasive listeriosis is less severe, where individuals may be asymptomatic or experience mild influenza‐like symptoms such as fever and muscle aches, whereas invasive listeriosis occurs when *L. monocytogenes* crosses the blood–brain and placental barriers, potentially leading to adverse pregnancy outcomes including fetal meningitis, miscarriage, stillbirth, or fetal death [4].

Pregnant women are 20 times more likely to contract listeriosis compared with the general population [5]. This is due to the suppressed cell‐mediated immunity from hormonal changes during pregnancy, with the placenta providing a protective environment that favors the growth of the bacterium [6, 7]. A systematic review in 2010 identified that pregnancy‐associated listeriosis accounted for 20.7% of listeriosis cases worldwide, with a fatality rate of 14.9% [8]. Therefore, prevention of listeriosis among pregnant women is critical to reduce the risk of adverse pregnancy outcomes and neonatal complications [9–11]. Internationally recognized guidelines have been established to reduce the risk of listeriosis in at‐risk populations including pregnant women [2]. In Australia, the Food Standards Australia New Zealand (FSANZ) provide dietary recommendations and guidelines for safe food handling, preparation, and storage to reduce the risk of listeriosis in vulnerable populations including pregnant women [12].

Previously, a secondary analysis of data from the Australian Longitudinal Study on Women′s Health, in women aged 25–30 years (*n* = 7486) trying to conceive, currently pregnant, or having delivered a baby in the previous year, found that higher consumption of foods potentially harboring *L. monocytogenes* was associated with a 19% higher rate of miscarriage after adjusting for potential confounders [13]. The birth outcomes in this study, however, conducted over a decade ago, were self‐reported, which could lead to inaccuracies in reporting as women can only report on recognized pregnancy outcomes while unable to report on early conceptual losses that may have gone undetected [14, 15]. Additionally, given the shifts in dietary patterns over the past decade [16, 17], updated research is needed to reflect more recent assessments of dietary intake related to potential *L. monocytogenes* exposure. Further, although pregnant women are advised to avoid high‐risk foods to reduce the risk of adverse events such as miscarriage and stillbirth, a recent study also found that this may unintendedly impact the intake of essential nutrients provided by these foods, resulting in nutritional inadequacy [18]. As such, it is important to examine the potential effects of *L. monocytogenes* contamination through dietary intake on perinatal outcomes and the associated hospital resource use, to better inform dietary practices during pregnancy while also limiting the risk associated with listeriosis. The current study is aimed at addressing this gap by evaluating associations between potential listeriosis exposure through food consumed during pregnancy and (i) infant outcomes and (ii) infant hospital resource use in a contemporary sample of Australian mother–infant dyads.

## 2. Methods

### 2.1. Study Design

The current study is an analysis of data combined from two cohorts of pregnant women recruited via antenatal clinics in Australia. The protocols of both studies have been previously reported [19, 20].

### 2.2. Study Population

Participants included in the current analyses were women aged ≥ 19 years, with a singleton pregnancy, between 28 and 42 weeks of gestation, who attended the John Hunter Hospital (JHH) and Joondalup Health Campus (JHC) antenatal clinics for routine antenatal care and had complete dietary and infant outcomes data. Multiple gestations were excluded due to their distinct clinical risk profiles and obstetric management pathways, which may introduce heterogeneity unrelated to dietary exposure and influence infant outcomes and hospital resource use. All participants gave their written informed consent for inclusion in the study.

### 2.3. Study Recruitment

#### 2.3.1. JHH Participants

Participants from JHH were recruited between March and November 2018, via clinic waiting room flyers, media release, and social media posts on Hunter Medical Research Institute and University of Newcastle Facebook pages, as described in detail elsewhere [21, 22].

#### 2.3.2. JHC Participants

ORIGINS is a collaborative initiative between the JHC and The Kids Research Institute Australia, Western Australia to establish a longitudinal cohort of 10,000 pregnant women and their partners [23]. Participants from ORIGINS were recruited between January 2017 and December 2022 via the JHC by their maternity or postnatal care provider, or by a member of the research team, as described elsewhere [19, 24].

### 2.4. Data Collection

#### 2.4.1. Maternal Sociodemographic and Health Characteristics

Participants self‐reported their sociodemographic and health data including age, weeks of gestation (at survey completion), birth country, language spoken, education level, postcode, marital status, height, and pre‐pregnancy weight via an online survey during antenatal visits. Pre‐pregnancy BMI was calculated from the self‐reported height and weight. Parity and smoking status data were obtained from hospital medical records. Postcodes were cross‐referenced with the Index of Relative Socioeconomic Advantage and Disadvantage (IRSAD) from the Socioeconomic Indexes for Areas (SEIFA) indexes from the Australian Bureau of Statistics (ABS) to identify participants residing in areas of differing socioeconomic status [25]. A lower IRSAD score indicates relatively greater disadvantage and lack of advantage, whereas a higher score suggests a relative lack of disadvantage and greater advantage based on census data including income, education, employment, occupation, housing, and family structure.

#### 2.4.2. Dietary Intake

Usual dietary intake of participants was self‐reported between gestational weeks 28 and 42 using the Australian Eating Survey (AES), a validated semiquantitative food frequency questionnaire (FFQ) consisting of 120 questions including questions on beverages (e.g., alcoholic drinks), milk and dairy foods, breads and cereals, sweet and savory snacks, main meals, vegetables, fruit, and other foods [26]. The AES was also used to assess consumption of foods included in the questionnaire that may potentially harbor *L. monocytogenes*. In addition, the AES contains 15 supporting questions about age, use of nutrition supplements, and intake‐related behaviors (e.g. frequency of takeaway meals, consumption of breakfast, and eating front of television) [26]. The AES assesses usual consumption frequency over the previous 3–6 months. Responses categories use a Likert Scale with options ranging from “never” to “four or more times per day,” and for some beverages up to “seven times per day.” Standard portion sizes for each food item in the AES were derived from the most recent National Nutrition Survey data [27]. Nutrient intakes from the AES FFQ were computed using data in the AUSNUT 2011–13 database [12]. The AES has been validated in the adult population and demonstrated high level of accuracy in estimating nutrient intake and fruit and vegetable intakes [28, 29]. It has also been previously used for measuring dietary intake of Australian pregnant women [30, 31], and in pregnancy cohort studies across Australia including ORIGINS [19], Baby1000 [32], Queensland Family Cohort [33], and NEW1000 [34].

#### 2.4.3. Potential *L. monocytogenes* Exposure

Potential *L. monocytogenes* exposure was quantified using the *Listeria* Food Exposure Score (LFES) (Table 1) based on the FSANZ public health recommendations [12]. The LFES was developed based on 11 foods across six categories (vegetables, fruit, dairy foods, meat, seafood, and delicatessen meats) within the AES identified as potential sources of *L. monocytogenes* [12]. The LFES scored frequency of consumption of each food item based on responses from “never” (scored as zero) to “two or more times per day” (scored up to seven points). The maximum LFES was 57 points, with higher scores indicative of more frequent consumption of foods that potentially harbor *L. monocytogenes*. Table 1 describes the scoring breakdown of the frequency of consumption for each food item listed in the LFES. The options for response categories range from “never” with zero points to “two or more times per day” to a maximum of seven points.

**Table 1:** The breakdown of scoring of food items in the *Listeria* Food Exposure Score (LFES).

Food groups	Food items	Frequency (points)	Score range
Vegetables	Lettuce	Never (0)	0–7 points
		Less than 1 per month (1)	
		1–3 per month (2)	
		Once per week (3)	
		2–4 times per week (4)	
		5‐6 times per week (5)	
		Once per day (6)	
		2 or more times per day (7)	
	Spinach	Never (0)	0–5 points
		Less than 1 per month (1)	
		1–3 per month (2)	
		Once per week (3)	
		2–4 times per week (4)	
		5 or more per week (5)	
	Cabbage and Brussels sprouts	Never (0)	0–5 points
		Less than 1 per month (1)	
		1–3 per month (2)	
		Once per week (3)	
		2–4 times per week (4)	
		5 or more per week (5)	

Fruit	Melon	Never (0)	0–5 points
		Less than 1 per month (1)	
		1–3 per month (2)	
		Once per week (3)	
		2–4 times per week (4)	
		5 or more per week (5)	

Meat	Liver—beef, calf, and chicken (including meat spreads)	Never (0)	0–5 points
		Less than 1 per month (1)	
		1–3 per month (2)	
		Once per week (3)	
		2–4 times per week (4)	
		5 or more per week (5)	

Delicatessen meats	Devon/luncheon meat and salami	Never (0)	0–5 points
		Less than 1 per month (1)	
		1–3 per month (2)	
		Once per week (3)	
		2–4 times per week (4)	
		5 or more per week (5)	
	Bacon and ham	Never (0)	0–5 points
		Less than 1 per month (1)	
		1–3 per month (2)	
		Once per week (3)	
		2–4 times per week (4)	
		5 or more per week (5)	
	Sausages, Frankfurts/hot dog, and Pluto Pup/corn dog	Never (0)	0–5 points
		Less than 1 per month (1)	
		1–3 per month (2)	
		Once per week (3)	
		2–4 times per week (4)	
		5 or more per week (5)	

Seafood	Other seafood (e.g. prawns/shrimp and lobster)	Never (0)	0–5 points
		Less than 1 per month (1)	
		1–3 per month (2)	
		Once per week (3)	
		2–4 times per week (4)	
		5 or more per week (5)	

Dairy	Ice cream—vanilla, chocolate, strawberry	Never (0)	0–6 points
		Less than 1 per month (1)	
		1–3 per month (2)	
		Once per week (3)	
		2–6 times per week (4)	
		Once per day (5)	
		2 or more per day (6)	
	Cottage cheese or ricotta	Never (0)	0–4 points
		Less than 1 per month (1)	
		1–3 per month (2)	
		Once per week (3)	
		2 or more per week (4)	

#### 2.4.4. Infant Outcomes and Hospital Resource Use

Infant outcomes and hospital resource use were extracted from JHH eMaternity and JHC Midwives′ Notification System electronic medical records databases. eMaternity is a clinical database used in the Hunter New England Local Health District to record maternal and infant health data during clinical care across the antenatal, intrapartum, and immediate postnatal periods [35], whereas the Midwives′ Notification System contains maternal and infant health data in Western Australia [36]. The systems captured data in a similar way allowing the datasets to be merged, and the following outcomes to be analyzed. The infant outcomes included were infant mode of birth, preterm birth, low birthweight, macrosomia at birth, small‐for‐gestational‐age, large‐for‐gestational‐age, and admissions to either special care nursery (SCN) or neonatal intensive care unit (NICU). Infant mode of birth was categorized as vaginal, cesarean, or instrumental birth. Preterm birth was defined as birth at less than 37 weeks [37]. Birthweight less than 2500 g was classified as low birthweight, and birthweight > 4000 g classified as macrosomia [38]. Birthweight above the 90th percentile for gestational age was classified as large‐for‐gestational‐age and < 10th percentile as small‐for‐gestational‐age [39].

For hospital resource use, the following data were included (i) infant overall (length of stay) LOS in hospital, which is measured by the difference between the number of days from the recorded date of birth and infant discharge date; (ii) infant LOS excluding time spent in SCN or NICU was calculated as the number of days the infant spent in hospital excluding time spent in NICU/SCN; and (iii) infant LOS in either SCN or NICU. The infant LOS excluding time spent in SCN or NICU was based on the recorded infant LOS in both datasets and intended to represent any potential adverse events on the mother′s health suggested by an increased LOS.

### 2.5. Statistical Analysis

Data were analyzed using STATA 18.0. [40] and R v.3.3.1 [41]. Participant sociodemographic, health, and dietary characteristics were summarized as mean (standard deviation) or median (interquartile range) for continuous variables (age, gestation weeks at survey completion, and birth gestation), and count (percentages) for categorical variables (parity, country of birth, language, education level, marital status, pre‐pregnancy BMI category, and smoking status). As a descriptive and exploratory study, a formal power calculation was not conducted. Complete case analysis was used, with analytic sample sizes ranging from 1175 to 1204 participants depending on the outcome. This sample size provided good precision for estimating associations, allowing detection of odds ratios (OR) with margins of error between ±0.38 and ±0.69 for rare outcomes with prevalence ranging from 5% to 15%, and rate ratios with margins of error of approximately ±0.07 at a 95% confidence level. This precision is reflected in the confidence intervals observed throughout the regression analyses (Tables 2, 3, and 4). Multinomial logistic regression analyses were conducted to investigate the association between LFES and infant mode of birth, categorized as vaginal, instrumental, or cesarean. Logistic regression analyses were used to measure the relationship between LFES and infant birth outcomes including preterm birth, low birth weight, small or large for gestational age, macrosomia, and any admission to NICU and/or SCN. The linearity assumption for continuous predictors was assessed by plotting the predictor (LFES) against the logit of the outcomes. All models were adjusted for covariates including smoking status, parity, maternal age, BMI, and SEIFA IRSAD decile [42, 43]. Results of both adjusted and unadjusted regression models were provided as a comparative analysis and reported as OR. Model fit was evaluated using the Hosmer–Lemeshow test and visual analysis of residual plots. Initial exploratory data analysis included the use of histograms to check the distribution of the data on infant hospital resource use. Due to right‐skewed distributions and overdispersion, the overall infant LOS and LOS excluding time spent in NICU/SCN were modeled through negative binomial regression models, using the MASS package [44]. Model fit was assessed using deviance residuals and pseudo *R* ^2^. For infant LOS in NICU/SCN, due to high zero counts, a negative binomial hurdle model was produced using the package glmmTMB [45]. The appropriateness of the hurdle model was confirmed by comparing AIC/BIC values with alternative models including a zero‐inflated negative binomial model. Results with a *p* value of < 0.05 were considered statistically significant. All statistical tests were two‐sided, and 95% confidence intervals are reported alongside point estimates.

**Table 2:** Regression analyses of potential *Listeria monocytogenes* exposure (LFES) and infant mode of birth.

Mode of birth	Adjusted^b^ (*n* = 1204)			Unadjusted (*n* = 1385)		
	OR (95% CI)	*p* value∗	Pseudo *R* ^2^ ^c^	OR (95% CI)	*p* value∗	Pseudo *R* ^2^ ^c^
Vaginal^a^	—	—	0.07	—	—	0.001
Cesarean	0.98 (0.95, 1.01)	0.15		0.995 (0.97, 1.02)	0.62	
Instrumental	0.99 (0.94, 1.03)	0.51		0.995 (0.96, 1.03)	0.73	

Abbreviations: CI, confidence interval; OR, odds ratio.

^a^Reference category.

^b^Adjusted for smoking status, parity, maternal age, BMI, and SEIFA IRSAD decile.

^c^Pseudo *R* ^2^ is used to assess goodness of fit for logistic regression models.

∗*p* < 0.05 indicates statistical significance.

**Table 3:** Regression analyses of potential *Listeria monocytogenes* exposure (LFES) and infant birth outcomes.

Infant birth outcomes	Adjusted^b^			Unadjusted (*n* = 1604)		
	OR (95% CI)	*p* value∗	Pseudo *R* ^2^ ^c^	OR (95% CI)	*p* value∗	Pseudo *R* ^2^ ^c^
Preterm birth (*n* = 1190)^a^	0.99 (0.93, 1.05)	0.77	0.08	0.98 (0.93, 1.02)	0.30	0.002
Low birthweight (*n* = 1175)^a^	1.02 (0.94, 1.10)	0.68	0.12	1.01 (0.96, 1.08)	0.66	< 0.001
Small for gestational age (*n* = 1204)^a^	1.01 (0.95, 1.07)	0.88	0.10	0.99 (0.95, 1.05)	0.83	< 0.001
Large for gestational age (*n* = 1204)^a^	1.01 (0.98, 1.05)	0.54	0.04	0.99 (0.97, 1.02)	0.64	< 0.001
Macrosomia (*n* = 1204)^a^	1.02 (0.97, 1.06)	0.51	0.04	1.02 (1.0, 1.04)	0.12	0.001
Any NICU/SCN admission (*n* = 1204)^a^	1.03 (0.99, 1.07)	0.11	0.05	1.02 (1.0, 1.05)	0.12	0.002

Abbreviations: CI, confidence interval; NICU, neonatal intensive care unit; OR, odds ratio; SCN, special care nursery.

^a^Sample size for adjusted analyses.

^b^Adjusted for smoking status, parity, maternal age, BMI, and SEIFA IRSAD decile.

^c^Pseudo *R* ^2^ is used to goodness of fit for logistic regression models.

∗*p* < 0.05 indicates statistical significance.

**Table 4:** Potential *Listeria monocytogenes* exposure (LFES) predicting infant total LOS and infant LOS excluding time spent in NICU/SCN.

Infant hospital resource use	Adjusted^a^		Unadjusted	
	Exp(*β*) (95% CI)	*p* value∗	Exp(*β*) (95% CI)	*p* value∗
Infant total LOS (days) (*n* = 1204)	0.99 (0.98, 1.00)	0.10	1.00 (0.99, 1.004)	0.50
Infant LOS excluding time spent in NICU/SCN (days) (*n* = 1181)^b^	0.99 (0.979, 0.998)	**0.03**	1.00 (0.99, 1.01)	0.95

Abbreviations: CI, confidence interval; Exp(*β*), exponentiated coefficient; LOS, length of stay; NICU, neonatal intensive care unit; SCN, special care nursery.

^a^Adjusted for smoking status, parity, maternal age, BMI, and SEIFA IRSAD decile.

^b^Twenty‐three participants had a negative length of stay, which was likely due to errors in recording; hence, this variable was set to be missing for 23 participants.

∗*p* < 0.05 indicates statistical significance and in bold.

## 3. Results

A total of 1604 mother–infant dyads with complete data were included in this study. Of these, 1201 were from the JHC and 403 were from the JHH.

Table 5 summarizes participant characteristics and LFES. Mean (SD) age of participants was 32.0 (5.0) years. The median (IQR) of gestation weeks at survey completion was 35 (34, 36) weeks, whereas the birth gestation was 39.0 (38.1, 40.0) weeks. Almost half of the women (43.1%) reported being pregnant with their first child. Most women were born in Australia (57.1%), spoke English at home (76.3%), and were married or in a de facto relationship (82.0%). In terms of socioeconomic status, the greatest proportion of participants (31.7%) resided in SEIFA IRSAD Quintiles 7–8, which indicates a profile of more advantaged and fewer disadvantaged economic and social conditions. Thirty percentage of participants held a university degree. Pre‐pregnancy BMI was calculated for 1264 participants, and 35.5% were classified as having a healthy BMI, whereas 41% were classified as having overweight (19.6%) or obesity (21.4%). In total, 4.2% women reported smoking during pregnancy. The mean (SD) LFES score was 16.4 (5.2) out of a maximum of 57 points.

**Table 5:** Sociodemographic characteristics and LFES of participants (*n* = 1604).

Variable	Value
Age (years), mean (SD)	32.0 (5.0)
Gestational weeks at survey completion, median (IQR)	35 (34, 36)
Missing	241
Birth gestation^a^ (weeks), median (IQR)	39.0 (38.1, 40.0)
Missing	219
Parity, *n* (%)	
0	692 (43.1)
1	450 (28.1)
2	175 (10.9)
≥ 3	65 (4.1)
Missing	222 (13.8)
Born in Australia, *n* (%)	
Yes	916 (57.1)
No	454 (28.3)
Missing	234 (14.6)
Language spoken at home, *n* (%)	
English	1224 (76.3)
Other	147 (9.2)
Missing	233 (14.5)
Highest education level, *n* (%)	
University degree	493 (30.7)
Trade certificate or diploma	305 (19.0)
High school certificate (Years 10–12)	319 (19.9)
Other/no formal qualifications	62 (3.9)
Missing	425 (26.5)
SEIFA IRSAD quintile, *n* (%)	
1–2	100 (6.2)
3–4	129 (8.0)
5–6	390 (24.3)
7–8	509 (31.7)
9–10	254 (15.8)
Missing	222 (13.8)
Marital status, *n* (%)	
Married/de facto	1315 (82.0)
Never married	41 (2.6)
Divorced/separated	22 (1.4)
Not reported/answered	7 (0.4)
Missing	219 (13.7)
Pre‐pregnancy BMI category (kg/m^2^), *n* (%)	
Underweight (< 18.5)	38 (2.4)
Healthy weight (≥ 18.5 to ≤ 24.9)	569 (35.5)
Overweight (≥ 25 to ≤ 29.9)	314 (19.6)
Obese (Classes I–III) (≥ 30 to ≥ 40)	343 (21.4)
Missing	340 (21.2)
Smoking during pregnancy, *n* (%)	
No	1318 (82.2)
Yes	67 (4.2)
Missing	218 (13.6)
LFES (0–57 points), mean (SD)	16.4 (5.2)

*Note:* SEIFA IRSAD Quintiles 9–10 indicates the most advantaged and least disadvantaged areas, whereas 1–2 indicated the least advantaged and most disadvantaged areas.

Abbreviations: BMI, body mass index; IRSAD, the Index of Relative Socioeconomic Advantage and Disadvantage; LFES, *Listeria* Food Exposure Score; SEIFA, Socioeconomic Indexes of Areas.

^a^Gestational age at birth.

Table 3 presents the adjusted and unadjusted logistic regression results using LFES as a predictor of infant birth outcomes. Overall, there was no statistically significant association between LFES and: preterm birth (*A* *d* *j*.*OR* = 0.99, 95% CI: 0.93, 1.05, *p* = 0.77); low birthweight (*A* *d* *j*.*OR* = 1.02, 95% CI: 0.94, 1.10, *p* = 0.68); small for gestational age (*A* *d* *j*.*OR* = 1.01, 95% CI: 0.95, 1.07, *p* = 0.88); large for gestational age (*A* *d* *j*.*OR* = 1.01, 95% CI: 0.98, 1.05, *p* = 0.54); macrosomia (*A* *d* *j*.*OR* = 1.02, 95% CI: 0.97, 1.06, *p* = 0.51); or any NICU/SCN admission (*A* *d* *j*.*OR* = 1.03, 95% CI: 0.99, 1.07, *p* = 0.11).

Table 2 presents the adjusted and unadjusted multinomial logistic regression results with LFES as a predictor of infant mode of birth. In the adjusted model, there were no statistically significant associations between LFES and the likelihood of cesarean (*A* *d* *j*.*OR* = 0.98, 95% CI: 0.95, 1.01, *p* = 0.15) or instrumental birth (*A* *d* *j*.*OR* = 0.99, 95% CI: 0.94, 1.03, *p* = 0.51), relative to vaginal birth.

Infant hospital resource use data is summarized in Table 6. Among 1385 infants, the median total LOS in hospital was 3 days (IQR: 2–4), with a mean (SD) of 3.21 (3.34) days. The time spent in NICU/SCN was minimal, with a median of 0 days (IQR: 0–0) and a mean (SD) of 0.42 (2.09) days. When excluding time spent in NICU/SCN, the median LOS remained 3 days (IQR: 2–4) among 1362 infants, with a mean (SD) of 3.10 (3.08) days.

**Table 6:** Infant hospital resource use (*n* = 1385).

Infant hospital resource use	Median (IQR)	Mean (SD)
Infant total LOS in hospital (days) (*n* = 1385)	3 (2–4)	3.21 (3.34)
Infant LOS in NICU/SCN (days) (*n* = 1385)	0 (0)	0.42 (2.09)
Infant LOS excluding time spent in NICU/SCN (days) (*n* = 1362)	3 (2–4)	3.1 (3.08)

Abbreviations: LOS, length of stay; NICU, neonatal intensive care unit; SCN, special care nursery.

Table 4 reports the adjusted and unadjusted negative binomial regression models for infant hospital resource use with LFES as a predictor. The adjusted analyses demonstrated that LFES was statistically significantly associated with infant LOS in hospital excluding time spent in NICU or SCN (*β* = 0.99; 95% CI: 0.979, 0.998, *p* < 0.03), indicating that for each one‐point increase in LFES, the expected infant LOS (excluding NICU/SCN) decreases by 1%. LFES was not statistically significantly associated with infant total LOS (*β* = 0.99, 95% CI: 0.98, 1.00, *p* < 0.10).

Table 7 reports the adjusted and unadjusted hurdle models for LFES as a predictor of infant LOS in the NICU or SCN. In both the adjusted conditional model and zero‐inflated model, the LFES was not statistically significantly associated with the infant′s LOS in the NICU/SCN (*A* *d* *j*.*β* = 1.01, 95% CI: 0.98, 1.05, *p* = 0.46) and (*A* *d* *j*.*β* = 0.99, 95% CI: 0.95, 1.03, *p* = 0.56), respectively.

**Table 7:** Potential *Listeria monocytogenes* exposure (LFES) predicting infant LOS in NICU/SCN (*n* = 1198).

Infant LOS in NICU/SCN (days)	Adjusted^a^		Unadjusted	
	Exp(*β*) (95% CI)	*p* value∗	Exp(*β*) (95% CI)	*p* value∗
Conditional model (rate ratio)	1.01 (0.98, 1.05)	0.46	0.98 (0.94, 1.01)	0.23
Zero‐inflated model (odds ratio)	0.99 (0.95, 1.03)	0.56	0.98 (0.95, 1.01)	0.20

Abbreviations: CI, confidence interval; Exp(*β*), exponentiated coefficient; LOS, length of stay.

^a^Adjusted for parity, maternal age, BMI, smoking during pregnancy, and SEIFA IRSAD decile.

∗*p* < 0.05 indicates statistical significance.

## 4. Discussion

The current study aimed to identify associations between potential *L. monocytogenes* exposure during pregnancy and infant outcomes and hospital resource use. This study ound that higher frequency of consumption of foods potentially harboring *L. monocytogenes* was associated with marginally shorter infant LOS in hospital, excluding time spent in NICU or SCN. However, there were no significant associations between consumption frequency of foods potentially harboring *L. monocytogenes* and infant mode of birth, preterm birth, low birthweight, macrosomia at birth, small‐for‐gestational‐age, large‐for‐gestational‐age, admissions to either SCN or NICU, infant total LOS in hospital, or infant LOS in either SCN or NICU.

In examining the association between the LFES and infant hospital resource use, a higher LFES was associated with a minor reduction in infants′ postbirth hospital stay, excluding NICU/SCN duration. This finding may suggest that infants of women with higher LFES, indicating a higher frequency of consuming foods potentially harboring *L. monocytogenes*, experience fewer health complications that require hospitalization post birth. This could be related to our recent research in a similar sample of pregnant women, which found that more frequent consumption of foods potentially harboring *L. monocytogenes* was significantly associated with higher diet quality characterized by high consumption of vegetables, fruits, whole grains, fish, and dairy products (*r* = 0.60, *p* < 0.001) [18]. Further, higher diet quality is also associated with lower risk of preterm birth, stillbirth, and low birth weight babies [46, 47]. This suggests that women with a higher LFES are likely to have a higher diet quality and lower risk of adverse birth outcomes, which could shorten infant LOS excluding time spent in NICU/SCN. However, it is important to acknowledge that the observed association is minor, limiting clinical significance. The narrow confidence intervals (*β* = 0.99; 95% CI: 0.979, 0.998, *p* < 0.03) allowed detection of a 1% decrease in LOS per LFES point, which although statistically significant, may not represent a clinically meaningful difference [48]. Further, the large sample size of this descriptive secondary analysis (*n* = 1604) may have increased statistical power to detect minor differences [49]. Therefore, the limited significance warrants the need for further research to evaluate whether maternal consumption of foods potentially harboring *L. monocytogenes* is associated with clinically meaningful differences in infant health outcomes and hospital resource use.

The current study found that the potential exposure to *L. monocytogenes*, measured by the LFES, is not statistically significantly associated with any of the infant birth outcomes, even after adjusting for potentially confounding factors. The sample mean (SD) LFES of 16.4 (5.2) points out of 57 indicates relatively low consumption of foods potentially harboring *L. monocytogenes* among participants. The SD of 5.2 reflects limited variability in the scores, which could limit the ability to detect significant relationships due to insufficient variation in the consumption of such foods. Further, consistent with previous research [13], low to moderate intake of foods potentially containing *L. monocytogenes* was not associated with increased risk of adverse birth outcomes including miscarriage, stillbirth, and preterm birth. This suggests that the low sample mean LFES may imply a reduced risk of listeriosis infection. However, both the present and previous studies did not assess food safety practices, making it unclear whether the lack of significant associations observed in this sample was due to dietary avoidance alone or in combination with proper food handling. Therefore, future research should consider integrating food safety assessments alongside dietary intake evaluations at recruitment. This approach would provide more comprehensive evidence on whether the frequency of consuming foods that may harbor *L. monocytogenes*, combined with food safety practices, influences infant birth outcomes and infant delivery resource use.

It is important to consider potential reasons why no other significant relationships were observed between potential *L. monocytogenes* exposure during pregnancy and infant outcomes or hospital resource use. This could be due to the period in which pregnant women were recruited and self‐reported their dietary intake. Listeriosis infections that occur in early gestation generally have poorer outcomes including stillbirth or spontaneous abortion compared with those that occur later in pregnancy [50]. The pregnant women that participated in this study were recruited in the third trimester (i.e., median of 35 weeks). As such, women who experienced adverse maternal outcomes such as miscarriage and preterm birth prior were not recruited into this study [51]. To better understand these associations, future research may need to focus on recruiting women from earlier stages of pregnancy, where the risk of severe adverse outcomes such as miscarriage is higher.

The next factor that could contribute to the lack of significant relationships in this study is the type of strains present in different food groups. Specific strains of *L. monocytogenes* are associated with consumption of different food groups and this could lead to different listeriosis outcomes [52]. Previous research reported that the hypervirulent strain of *L. monocytogenes* is highly distributed in dairy food products as compared with other food groups, and is more adept at colonizing human intestinal cells, leading to invasive listeriosis [53]. In our recent cohort study of pregnant women in Australia (*n* = 1638), with a similar LFES mean score (16.3) and gestational age at dietary intake collection (35 weeks), LFES showed the lowest correlation with dairy foods intake (*r* = 0.11, *p* < 0.001), compared with other food groups (*r* = 0.15–0.43, *p* < 0.001) [18], indicating a low consumption of dairy foods. This suggests that the current sample may also have low intake of dairy food groups, and therefore, reduced risk of contracting invasive listeriosis, potentially contributing to the lack of significant findings. Given the higher risk associated with dairy food products and the variation in *L. monocytogenes* strains across different food groups, future studies should consider dairy food intake when examining the association with perinatal outcomes.

Another factor that may have contributed to the lack of significant associations observed in the current study is the level of food safety knowledge among pregnant women. Adequate knowledge of food handling is critical to preventing listeriosis, particularly in vulnerable populations such as pregnant women and especially first‐time mothers [54, 55]. An umbrella review identified a lack of knowledge as a significant factor contributing to listeriosis risk during pregnancy [9]. Previous studies also found that first‐time mothers exhibited significantly higher food safety knowledge scores [56], and they are likely to have greater adherence to food safety guidelines [57]. This suggests that first‐time mothers may be more vigilant about their dietary intake and practices that could influence their health and the health of their infant. In our study, the majority of participants were first‐time mothers with lower intake of foods potentially harboring *L. monocytogenes*, as indicated by a mean LFES of 16.4 out of 57 points. This reduced exposure could explain the lack of significant associations between LFES and infant outcomes observed in the current study.

Moreover, ready‐to‐eat (RTE) foods are linked to supporting the growth of *L. monocytogenes*, which increases its risk of contributing to listeriosis [58]. Microbiological testing for *L. monocytogenes* is therefore a critical safety measure to detect the presence of the bacteria in food. In Australia, FSANZ sets mandatory microbiological limits for *L. monocytogenes* in RTE foods to minimize consumer exposure to *L. monocytogenes* and reduce the likelihood that ingested doses reach levels capable of causing infection [59]. In the data collection tool (LFES), delicatessen meats and pâté items can be considered as RTE foods [12]. However, based on the FSANZ decision framework and Codex recommendations [58, 60], foods that meet predefined pH and water activity criteria may not require microbiological testing. This study did not collect detailed information on the specific types of food products consumed by participants and therefore lacks data on their pH and water activity levels. Future studies could refine data collection to capture these details to allow for more precise exposure assessment of the association between LFES and infant outcomes in the current study.

### 4.1. Strengths and Limitations

The current study has several strengths, including the large sample of Australian pregnant women recruited from two antenatal health clinics. Next, the sociodemographic characteristics of participants in the current analysis are broadly reflective of the Australian population. Specifically, the distributions of maternal age, country of birth, and BMI categories were comparable with the latest national data [61]. For example, the proportion of women aged within the corresponding maternal age group was 31.3% in the study sample versus 32.0% nationally; mothers born in Australia accounted for 65% of the sample compared with 57.1% nationally; and the proportions classified as obese (21.6% vs. 21.4%) and overweight (25.0% vs. 19.6%) were also similar. Additionally, the median LOS of infants in hospital in the current sample (2–4 days) is comparable with national data, with the most common LOS for babies ranging between 2 and 3 days [62]. Next, the LFES was developed based on a contemporary list of higher risk food sources of *L. monocytogenes* from FSANZ [12] within a validated FFQ [26]. Further, the study adopted robust statistical methods, including adjustment for covariates. However, the following limitations need to be considered when interpreting the findings. Firstly, the self‐reported FFQ responses used to evaluate LFES and AES are susceptible to recall bias [63], although this does not limit the importance of data information collected about dietary intake [64]. Although the LFES was developed based on evidence‐based public health FSANZ recommendation, aimed at preventing foodborne illnesses in pregnant women [12], it has not been validated and does not capture key food safety behaviors, including food storage, reheating, and hygiene practices. These behaviors substantially influence actual *L. monocytogenes* exposure risk, meaning their absence may have led to exposure misclassification and biased effect estimates toward the null. As a result, the findings, particularly for null or small associations, should be interpreted with caution. Future research should focus on validating the LFES and integrating the assessment of food safety practice tailored to pregnant women to improve the accuracy and reliability of the LFES in measuring the risk of listeriosis infection, subsequently providing more robust data for analysis. Additionally, the timing of dietary data collected should be considered when interpreting these findings. The LFES was developed using items from the AES in accordance with FSANZ evidence‐based guidelines current at the time of study conduct, which identified high‐risk food categories including vegetables, fruits, dairy products, meat, seafood, and delicatessen meats [12]. Although more recent FSANZ updates (October 2025) listed additional specific items [65], these were released after data collection (2017–2022) and hence incorporated into the scoring system at the time the study was concluded. However, these items fall within existing LFES food categories and are unlikely to alter exposure classification at the category level. Therefore, findings derived from this study for assessing current dietary practices and listeriosis risk should be interpreted with this context in mind. Next, this study did not collect data on women diagnosed with listeriosis, their use of antibiotics, or outcomes of infants infected with listeriosis requiring SCN/NICU admission. As a result, the ability to precisely assess the relationship between consumption of foods potentially harboring *L. monocytogenes* and adverse perinatal outcomes is limited. Future studies should address this gap by including laboratory‐confirmed infections, antibiotic use, and relevant infant outcomes. Incorporating these data would strengthen the precision and accuracy of analysis regarding the exposure–outcome associations. Further, although the study was able to adjust for potential confounding factors associated with sociodemographic factors [42, 43], other known contributors to adverse pregnancy outcomes, including pregnancy‐related diabetes, were not considered due to incomplete data collection. Pregnancy‐related diabetes can influence outcomes such as gestational size at birth, birthweight, and long‐term health consequences for the child [66]. Future studies should prioritize consistent collection of maternal health data to enable broader and more robust analyses. Finally, although the sample from which the data have been drawn is large, the sample of women in the current study is predominantly Australian‐born, and data on participant ethnicity were not collected consistently across the two cohorts. As a result, the representativeness of the study population is limited, particularly given evidence that pregnancy‐associated listeriosis occurs more frequently among people of Pacific, Asian, and Hispanic ethnic backgrounds [57, 67]. To provide additional context, Table S1 summarizes the distribution of participants′ country of birth as a partial proxy for cultural or ethnic background. Future studies should be aimed at recruiting participants from diverse ethnic groups and collecting detailed ethnicity information to better reflect the Australian population and enable examination of ethnicity‐related differences in listeriosis risk.

## 5. Conclusion

In conclusion, among this sample of Australian pregnant women, greater frequency of consumption of foods that potentially harbor *L. monocytogenes,* as measured by the LFES, was associated with a small, but statistically significant reduction in infant hospital LOS, excluding time spent in the NICU or SCN. However, the magnitude of this association is unlikely to be clinically significant. No significant associations were found between the LFES and infant birth mode, birth outcomes, total LOS in hospital, and the LOS and odds of admission to the NICU/SCN. Future studies should examine the relationship between potential listeriosis exposure during pregnancy, perinatal outcomes, and hospital resource use by recruiting women earlier in pregnancy from diverse ethnic backgrounds. Consideration should also be given to assessing food safety practices to better inform contemporary and achievable dietary practices during pregnancy, while simultaneously reducing the risk of pregnancy‐associated listeriosis.

## Author Contributions

Clare E. Collins, Melinda Hutchesson, Rachael Taylor, and Sasha Fenton contributed to study conceptualization; Clare E. Collins, Melinda Hutchesson, Rachael Taylor, and Sasha Fenton obtained funding for the study; Sasha Fenton and Madeleine Hinwood developed the analysis plan; Madeleine Hinwood performed statistical analysis and provided statistical advice; Kee June Ooi prepared the draft manuscript. All authors contributed to the interpretation of study results and critical revision of the manuscript.

## Funding

This study was supported by National Health and Medical Research Council (10.13039/501100000925; APP2009340); Hunter Medical Research Institute (10.13039/501100001081); Telethon Perth Children′s Hospital Research Fund; Joondalup Health Campus; Paul Ramsay Foundation (10.13039/501100016053); and Commonwealth Government of Australia.

## Disclosure

All authors approved the final version of the manuscript for submission. This study is a subproject of ORIGINS. This unique long‐term study, a collaboration between The Kids Research Institute Australia and Joondalup Health Campus, is one of the most comprehensive studies of pregnant women and their families in Australia to date, recruiting 10,000 families over a decade from the Joondalup and Wanneroo communities of Western Australia. A preprint has previously been published [68]. C.E.C. is supported by a National Health and Medical Research Council of Australia Research Fellowship (APP2009340). Statistical analysis was supported by the Hunter Medical Research Institute Food & Nutrition Program Pilot Grants for Research Projects. ORIGINS has received core funding support from the Telethon Perth Children′s Hospital Research Fund, Joondalup Health Campus, the Paul Ramsay Foundation, and the Commonwealth Government of Australia through the Channel 7 Telethon Trust. Substantial in‐kind support has been provided by The Kids Research Institute Australia and Joondalup Health Campus.

## Ethics Statement

Ethics approval was obtained from the Hunter New England Human Research Ethics Committee (2019/ETH00954) and is registered with the University of Newcastle Human Research Ethics Committee (H‐2023‐0239) for the JHH cohort in New South Wales, Australia and the JHC Human Research Ethics Committee cohort (2023/ETH/0001) for the ORIGINS cohort in Western Australia, Australia.

## Conflicts of Interest

The authors declare no conflicts of interest.

## Supporting information


**Supporting Information** Additional supporting information can be found online in the Supporting Information section. Table S1: Frequency distribution of birth countries of participants (*n* = 1370).

## Data Availability

The data that support the findings of this study are available on request from the corresponding author. The data are not publicly available due to privacy or ethical restrictions.
