# Supplementary material for: Associations Between Potential Listeria monocytogenes Exposure During Pregnancy and Infant Outcomes at Birth and Infant Delivery Resource Use: A Cross‐Sectional Analysis in Australian Women
Source: J Pregnancy. 2026 Jul 31;2026:7824916. doi: 10.1155/jp/7824916 (PMC13428182; doi:10.1155/jp/7824916)
Supplement: Supplementary file 1 — Supporting Information Additional supporting information can be found online in the Supporting Information section. Table S1: Frequency distribution of birth countries of participants (n = 1370). [file JP-2026-7824916-s001.docx]

**Supplementary Table 1: Frequency distribution of birth countries of participants (*n* = 1,370)**

| **Birth Country** | **Frequency** |
| --- | --- |
| Australia | 916 |
| United Kingdom of Great Britain and Northern Ireland | 145 |
| New Zealand | 46 |
| South Africa | 42 |
| Ireland | 30 |
| India | 12 |
| England | 9 |
| Germany | 9 |
| Malaysia | 9 |
| Kenya | 8 |
| Brazil | 7 |
| China | 7 |
| Canada | 6 |
| France | 6 |
| United States of America | 6 |
| Indonesia | 5 |
| Philippines | 5 |
| Singapore | 5 |
| Taiwan, Province of China | 5 |
| Zimbabwe | 5 |
| Italy | 4 |
| Thailand | 4 |
| Colombia | 3 |
| Hong Kong | 3 |
| Japan | 3 |
| Nepal | 3 |
| Mexico | 2 |
| Papua New Guinea | 2 |
| Romania | 2 |
| Russian Federation | 2 |
| Slovakia | 2 |
| Switzerland | 2 |
| Ukraine | 2 |
| Vietnam | 2 |
| Botswana | 1 |
| Cambodia | 1 |
| Chile | 1 |
| Croatia | 1 |
| Egypt | 1 |
| El Salvador | 1 |
| Estonia | 1 |
| Eswatini | 1 |
| Finland | 1 |
| French Southern Territories | 1 |
| Greece | 1 |
| Hungary | 1 |
| Iceland | 1 |
| Korea (Democratic People's Republic of) | 1 |
| Korea, Republic of | 1 |
| Kuwait | 1 |
| Mauritius | 1 |
| Myanmar | 1 |
| Netherlands | 1 |
| North Macedonia | 1 |
| Pakistan | 1 |
| Peru | 1 |
| Poland | 1 |
| Saudi Arabia | 1 |
| Sri Lanka | 1 |
| Sweden | 1 |
| Venezuela (Bolivarian Republic of) | 1 |
| Zambia | 1 |
| Other | 23 |
| **Total** | **1,370** |
